# Immediate initiation of oral contraceptive pills after an early pregnancy loss does not increase the risk of retained products of conception

**DOI:** 10.3389/frph.2026.1918673

**Published:** 2026-09-11

**Authors:** Leah Cooper, Radhika Sharma, Mindy B. Frishman, Hanikka Muna, Danielle Jean, Yang Liu, Molly B. Moravek, Samantha B. Schon

**Affiliations:** 1 Reproductive Endocrinology and Infertility, Obstetrics & Gynecology, University of Michigan, Ann Arbor, MI, United States; 2 Department of Obstetrics & Gynecology, University of Michigan, Ann Arbor, MI, United States; 3 Florida State University, Tallahassee, FL, United States; 4 Department of Reproductive Medicine, Henry Ford Health, Rochester Hills, MI, United States

**Keywords:** early pregnancy loss, embryo transfer, infertility, office hysteroscopy, oral contraceptive pills, retained products of conception

## Abstract

**Introduction:**

Retained products of conception (rPOC) are a common complication following early pregnancy loss (EPL) and may contribute to infertility and delays in subsequent fertility treatment. Oral contraceptive pills (OCPs) are frequently initiated after EPL in infertility patients to synchronize the menstrual cycle prior to fertility treatment; however, it is unknown whether immediate initiation of OCPs affects the incidence of rPOC.

**Methods:**

We conducted a retrospective cohort study at a single academic reproductive endocrinology and infertility practice including patients who experienced EPL following embryo transfer between 2015 and 2023 and subsequently underwent office hysteroscopy (OHS). Patient demographics, infertility diagnoses, EPL management modality, and OCP initiation following resolution of EPL were collected through chart review. rPOC was diagnosed by direct visualization during OHS.

**Results:**

A total of 188 women with EPL following embryo transfer were included, of whom 42 (22.3%) were diagnosed with rPOC at the time of OHS. There were no significant differences in demographic or infertility characteristics between patients with and without rPOC. Following EPL, 52.1% of patients underwent surgical management, 31.9% expectant management, and 16.0% medical management, with no significant association between management modality and rPOC (*p* = 0.3). Fifty-five patients (29.3%) initiated OCPs following resolution of EPL. A history of uterine surgery before EPL was independently associated with rPOC. Patients who had prior uterine surgery were 2.38 times more likely to have rPOC than those who had no history of uterine surgery (aOR 2.38, 95% CI 1.08–5.73). OCP use was not associated with rPOC after adjusting for prior uterine surgery and time to OHS (aOR 1.17, 95% CI 0.54–2.47).

**Discussion:**

Immediate initiation of OCPs following EPL after embryo transfer was not associated with an increased risk of rPOC in this infertility population. These findings support the use of OCPs after EPL to facilitate fertility treatment planning, though larger prospective studies are needed to further evaluate factors associated with rPOC.

## 1 Introduction

Early pregnancy loss (EPL), commonly referred to as a miscarriage, is defined as a nonviable intrauterine pregnancy occurring within the first trimester. It can be diagnosed with ultrasound by observing an empty gestational sac or a gestational sac containing a fetus without cardiac activity (1–3). This is an unfortunately common phenomenon in reproductive aged women, occurring in 10%–25% of all clinically recognized pregnancies (2, 4). The incidence of EPL is higher with increasing maternal age. Despite advances in assisted reproductive technology (ART), women with infertility may also experience EPL after embryo transfer. EPL in this population frequently includes both clinical (visualized) and biochemical (non-visualized) pregnancies, given the early and close monitoring with serum beta human chorionic gonadotropin (hCG). Previous studies have found 5%–29% of pregnancies following embryo transfer result in an EPL (5–9).

The options for management of an EPL include expectant, medical or surgical management, and treatment choice is guided by the patient's clinical presentation and preferences (2, 3, 10). Following treatment of an EPL, a patient may have retained products of conception (rPOC), defined as retention of trophoblastic tissue inside the uterine cavity (11). A recent retrospective cohort study in 597 pregnancies resulting in EPL after embryo transfer reported retained products of conception in 21.6% of patients (12). Patients electing for medical management of EPL had the highest likelihood of rPOC (up to 53%) (12). Similar to the treatment options of EPL, the treatment of rPOC includes expectant, medical or surgical management. Given 30% of patients with rPOC may be asymptomatic, it is often incidentally found during follow up imaging (13, 14). The rate of complete evacuation of rPOC following expectant management is 47%–81% whereas after surgical management it is 95%–97% (11). rPOC can contribute to infertility and affect pregnancy rates in subsequent fertility treatment cycles if not diagnosed and managed. rPOC may also increase the time to conception, given the need for additional treatment (15).

After resolution of an EPL, infertility patients often begin oral contraceptive pills (OCPs) to synchronize their menstrual cycle for fertility treatments, such as another stimulation cycle or subsequent frozen embryo transfer cycle. One small pilot study examined the use of OCPs for medical management of rPOC and found 9 of 12 patients were successfully treated. The remaining patients who failed to resolve rPOC had vascular rPOC, suggesting that vascular adherent tissue may not detach with OCPs alone (16). It is also possible that the OCPs had no effect on the rate of successful resolution of rPOC, or that starting OCPs in the first menses after EPL is diagnosed could actually decrease the resolution of rPOC given the decrease in menses length and heaviness associated with OCP use. To our knowledge, no studies to date have examined the effects of OCP initiation immediately following EPL on the incidence of rPOC in an infertility population. Given the importance of optimizing time to conception and reducing intrauterine pathology, there is a critical gap in existing literature.

The primary objective of the current study was to determine if initiation of OCPs immediately following an EPL was associated with an increased prevalence of rPOC following an embryo transfer. Secondary objectives were to assess baseline patient characteristics and EPL treatment modalities associated with rPOC.

## 2 Methodology

**Figure 1:**
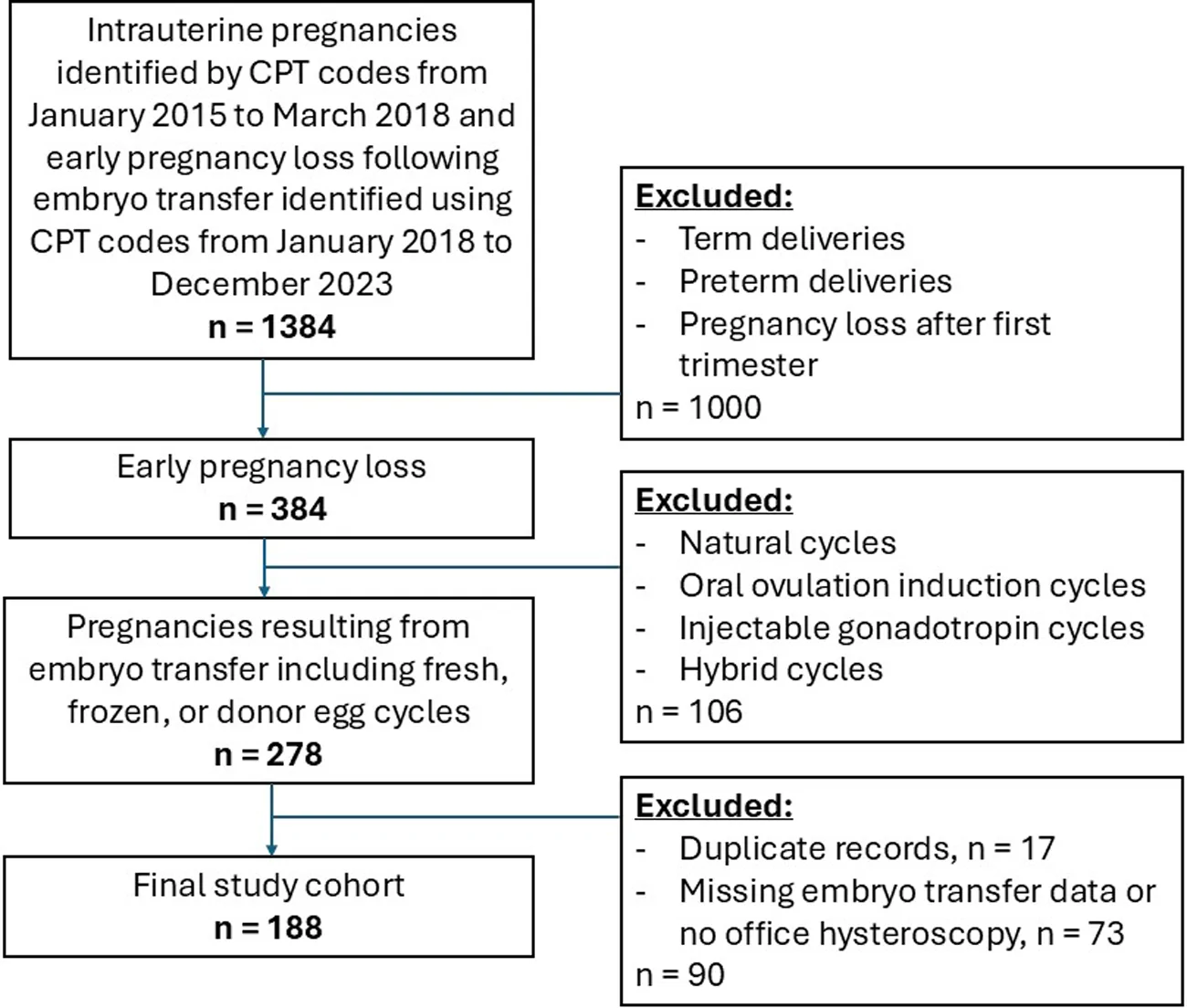
Patient selection flow diagram. The initial dataset included intrauterine pregnancies identified by CPT codes between January 2015 and March 2018. Additional patients who experienced early pregnancy loss following embryo transfer were identified using CPT codes from January 2018 through December 2023. Only patients who experienced early pregnancy loss following embryo transfer were included. The final study cohort included 188 patients.

We conducted a retrospective cohort study at a single academic reproductive endocrinology and infertility practice. Initial data was collected from a previous dataset which included over 1,200 pregnancies between January 2015 and March 2018 identified through current procedural terminology (CPT) codes to identify all obstetric ultrasounds performed following embryo transfer through methods previously described (17). Additional patients were added to the dataset after identification with similar CPT codes to include all patients who underwent embryo transfer resulting in an early pregnancy loss between January 2018 and December 2023 (Figure 1). Medical records were then reviewed to confirm individuals who experienced EPL following embryo transfer. Only patients who underwent subsequent uterine cavity evaluation with office hysteroscopy (OHS) were included in the study. Pregnancies of unknown location, ectopic or biochemical pregnancies were not included. Medical records were reviewed to collect patient demographics, including age, race, ethnicity, and body mass index (BMI). Additional clinical data extracted included infertility diagnosis, gynecologic and uterine surgical history prior to the recent EPL, embryo transfer details, EPL management, and whether patients initiated OCPs following negative beta hCG after resolution of EPL. Patients were only included in the analysis if they had a follow-up OHS prior to subsequent fertility treatment cycles. Time between embryo transfer that resulted in EPL and the OHS was calculated. The diagnosis of rPOC, defined as the presence of visible intrauterine tissue, was made at the time of OHS. All office hysteroscopies were performed by high volume board certified reproductive endocrinology and infertility specialists with expertise in the procedure and diagnosis of rPOC. Given the retrospective data collection for this study, providers were not blinded to the OCP exposure.

Statistical analysis performed included Wilcoxon rank sum test, Fisher's exact test, Chi-squared test, and a multivariable logistic regression as appropriate to determine associations between OCP use, patient demographics, infertility diagnosis, and EPL treatment with rPOC. Descriptive parameters are expressed in medians with interquartile range. Frequencies are given as percentages. If patients had missing data for their EPL and ET, they were excluded from the analysis. All demographic and clinic variables had complete data except race (*n* = 2 missing) and ethnicity (*n* = 3 missing). Analysis was performed using complete case analysis. All statistical analyses were carried out using R software (4.3.0). This study was determined to be exempt from full Institutional Review Board review.

## 3 Results

A total of 188 women diagnosed with EPL following embryo transfer were included in the analysis (Figure 1). 42 (22.3%) were found to have rPOC diagnosed at the time of OHS, while 146 had no evidence of rPOC. Patient demographics were comparable between the two groups (rPOC and non-rPOC) as shown in Table 1. There were no differences in age, race, or ethnicity between the two groups. The median age at embryo transfer was 35 years in both groups. The majority of patients were Caucasian, and non-Hispanic. The median BMI in the rPOC group was 25.0 kg/m^2^ versus 25.9 kg/m^2^ in the non-rPOC group. There were no significant differences in patient infertility diagnosis between the two groups. The most common infertility diagnosis in this cohort was ovulatory dysfunction (40.4%), which included polycystic ovary syndrome, diminished ovarian reserve, and hypothalamic amenorrhea. The second most common infertility diagnosis was male factor infertility (25.5%), followed by other diagnoses (15.4%). Most patients (73.9%) did not have a history of endometriosis, uterine polyps, or fibroids. There were no significant differences in gynecologic history between the two groups (*p* = 0.2). Only 3 patients in the non rPOC group had a uterine anomaly. Patient demographics and clinical variables for those individuals who did and did not start OCPs are demonstrated in Supplementary Table 1. Patients in the OCP use group were younger at transfer than the non-OCP group (median 34.8 [Interquartile range (IQR) 32.2, 37.1] vs. median 36.0 (IQR 32.4, 39.1), *p* = 0.049). There was no significant difference in race, ethnicity, BMI, baseline characteristics, EPL management modality, prior uterine surgery, time to OHS, or other relevant variables between the two groups. A multivariable logistic regression was utilized to investigate the relationship between OCPs and the risk of rPOC with the adjustment for covariates that were statistically significant in bivariate analysis as shown in Table 2. Prior uterine surgery was independently associated with rPOC. Patients who had prior uterine surgery were 2.38 times more likely to have rPOC than those who had no history of uterine surgery [Adjusted odds ratio (aOR) 2.38, 95% Confidence interval (CI) 1.08–5.73]. There was no statistically significant difference in the number of prior surgeries between patients with or without rPOC (*p* = 0.057).

**Table 1:** Demographics and characteristics.

Characteristic	Patients		*p*-value^b^
	rPOC	Non rPOC	
	*N* = 42^a^	*N* = 146^a^	
Age at embryo transfer	35.3 (32.5, 38.3)	35.6 (32.0, 38.5)	0.6
Race			>0.9
Caucasian	35 (83.3%)	113 (78.5%)	
Asian	4 (9.5%)	18 (12.5%)	
Other	2 (4.8%)	9 (6.3%)	
Black or African American	1 (2.4%)	4 (2.8%)	
Missing	0	2	
Ethnicity			0.7
Non-Hispanic	41 (97.6%)	135 (94.4%)	
Hispanic	1 (2.4%)	8 (5.6%)	
Missing	0	3	
BMI	25.0 (22.4, 29.5)	25.9 (23.0, 29.2)	0.6
Cause of infertility			0.3
Ovulatory dysfunction	16 (38.1%)	60 (41.1%)	
Male Factor	13 (31.0%)	35 (24.0%)	
Other	9 (21.4%)	20 (13.7%)	
Uterine factor	4 (9.5%)	21 (14.4%)	
Unexplained	0 (0.0%)	10 (6.8%)	
GYN diagnoses			0.2
None	30 (71.4%)	109 (74.7%)	
Endometriosis	4 (9.5%)	21 (14.4%)	
Endometrial Polyp	5 (11.9%)	8 (5.5%)	
Fibroid	2 (4.8%)	8 (5.5%)	
Other	1 (2.4%)	0 (0.0%)	
History of Asherman's syndrome	3 (7.1%)	5 (3.4%)	0.4
History of placenta accreta spectrum	0 (0.0%)	3 (2.1%)	>0.9
History of uterine surgery	33 (78.6%)	90 (61.6%)	0.042
Number of prior uterine surgeries (D&C, MVA, CS)			0.057
0	9 (21.4%)	56 (38.4%)	
1	12 (28.6%)	44 (30.1%)	
2	6 (14.3%)	20 (13.7%)	
≥3	15 (35.7%)	26 (17.8%)	
Uterine anomaly			>0.9
None	42 (100.0%)	143 (97.9%)	
History of septum	0 (0.0%)	1 (0.7%)	
Bicornuate	0 (0.0%)	1 (0.7%)	
Didelphys	0 (0.0%)	1 (0.7%)	
Time between ET^c^ and OHS (days)	100 (80, 115)	114 (79, 158)	0.042

a Median (Q1, Q3); *n* (%).

b Wilcoxon rank sum test; Fisher's exact test; Pearson's Chi-squared test.

c Embryo Transfer.

**Table 2:** Adjusted multivariable logistic regression model to investigate the relationship between OCP use and risk of rPOC.

Characteristic	aOR^a^	95% CI^a^	*p*-value
Started OCPs after resolution of pregnancy loss	1.17	0.54, 2.47	0.7
Prior uterine surgery	2.38	1.08, 5.73	0.039
Time to OHS	1.00	1.00, 1.00	0.6

a OR, adjusted odds ratio; CI, confidence interval.

After diagnosis of EPL, 98 women (52.1%) elected for surgical management, 30 (16.0%) elected medical management, and 60 (31.9%) elected for expectant management as shown in Table 3. There was no significant difference between EPL management modality and likelihood of rPOC (*p* = 0.3). The highest incidence of rPOC was in patients whose EPL was managed medically (33.3%), followed by 21.4% of patients managed surgically, and 18.3% of patients managed expectantly. The time between embryo transfer and OHS following EPL was significantly longer in the non rPOC group at 114 days [IQR 79–158] compared to 100 days [IQR 80–115] in the rPOC group (*p* = 0.042). Of the 188 women, 55 (29.3%) were started on OCPs after resolution of EPL defined by negative beta hCG. OCP use was not associated with rPOC after adjusting for prior uterine surgery and time to OHS (aOR 1.17, 95% CI 0.54–2.47) (Table 2).

**Table 3:** Management of EPL and OCP use.

Characteristic	EPL after embryo transfer			*p*-value^b^
	Overall *N* = 188^a^	rPOC *N* = 42^a^	Non rPOC *N* = 146^a^	
Miscarriage Treatment				0.3
Surgical	98 (52.1%)	21 (50.0%)	77 (52.7%)	
Expectant	60 (31.9%)	11 (26.2%)	49 (33.6%)	
Medical	30 (16.0%)	10 (23.8%)	20 (13.7%)	

a n (%).

b Pearson's Chi-squared test.

## 4 Discussion

In this retrospective cohort study of women who experienced EPL following embryo transfer, we found that the initiation of OCPs following negative beta hCG had no statistically significant association with the risk of rPOC. To our knowledge, this is the first study to explore the relationship between rPOC and OCP initiation, despite their common use in an infertility population to synchronize the menstrual cycle and expedite the time to subsequent fertility treatments.

Previous literature has examined the incidence of rPOC after different management strategies for EPL (12), as well as the efficacy of different treatments for rPOC (11, 18, 19). While these studies have shown the incidence of rPOC was higher following expectant or medical management of EPL, this study demonstrated a different numerical pattern, with the highest observed incidence of rPOC following medical or surgical management; however, these differences were not statistically significant and should be interpreted cautiously. The low incidence of rPOC (18.3%) following expectant management in this cohort may be the result of early and frequent monitoring in an infertility clinic where many of the patients who elected expectant management had incomplete or spontaneous abortions as opposed to a missed abortion at later gestations. The overall prevalence of rPOC in this cohort study (22.3%); as well as the prevalence of rPOC following medical (33.3%) and surgical (21.4%) management of EPL were comparable to previous literature. A recent study by Purusothaman et al. found rPOC rates after EPL to be 13.0% after manual vacuum aspiration compared to 29.4% after medical management (20). Similarly, in a retrospective cohort study of *in vitro* Fertilization (IVF)conceived pregnancies, rPOC was found in 21.6% of EPL cases through routine office hysteroscopy, with significantly higher rates following medical management compared to surgical intervention (adjusted RR 2.66, 95% CI 1.90–3.73) (12). In a separate study of rPOC after miscarriage, only 29% of women managed medically avoided subsequent surgical intervention (21). Our current study found no difference in the incidence of rPOC after different treatment options, however this may have been affected by our small sample size.

While earlier research has focused on the incidence of rPOC between different management methods, our study uniquely investigates whether the initiation of OCPs after EPL influences the risk of rPOC. Specifically, our study evaluated this in patients with an infertility diagnosis undergoing embryo transfer resulting in EPL, and determined if there were associations between OCP use, patient demographics, infertility diagnosis, and EPL treatment with rPOC. It is often common practice to initiate OCPs after the resolution of an EPL to expedite treatment planning and to synchronize the patient's menstrual cycle with fertility treatment. Our study did not identify a statistically significant association between the initiation of OCPs after EPL and rPOC, providing reassurance for their use in clinical practice, regardless of a patient's infertility diagnosis, or gynecological history. The current study contributes to the limited literature evaluating rPOC specifically in an infertility population following embryo transfer. The current study was exploratory and not adequately powered to detect a 10% difference in rPOC with OCP use due to small sample size. The absence of statistically significant association should not be interpreted as evidence of equivalence or safety. Rather these results suggest that any clinically important association remains uncertain and warrants evaluation in larger studies.

There was a significantly higher incidence of rPOC in patients with any history of prior uterine surgery compared to those who had not undergone uterine surgery. There was also a higher frequency of rPOC among patients who had undergone three or more uterine surgeries, although this was not statistically significant. This finding likely reflects the effects of intrauterine scarring or impaired endometrial healing after instrumentation. Prior literature has discussed a link between prior uterine surgery and rPOC. In a previous study, prior cesarean deliveries were identified as an independent risk factor for rPOC in placenta previa patients (22). Conversely, a retrospective cohort study of 132 live births after embryo transfer found rPOC were significantly more likely following vaginal delivery (23). Given prior literature linking uterine surgery to rPOC, this trend as it relates to OCP use warrants further research.

A major strength of this study was the standardized approach to use OHS following EPL to diagnose rPOC with direct visualization, which is more definitive and sensitive than ultrasound. Transvaginal ultrasound is often the first line imagining modality when rPOC are suspected but lacks specificity compared to OHS. Additionally, we captured a broad collection of demographics and clinically relevant factors for the evaluation of potential predictors and confounders. The clinical experience over 8 years provides practical, practice-informing estimates in an IVF population. This study is not without limitations. These results may not be generalizable to a more diverse or broad population given the limited study population from a single academic institution. The small sample size of this study may have limited our ability to detect statistical differences between the two groups with and without rPOC. Another limitation of this study was the inability to collect information about patient and provider decision-making in regard to modality of EPL management. Additionally, due to the retrospective data collection, time interval between negative beta hCG and OCP initiation, as well as duration of OCP use, was not readily available for all patients and not considered in our analysis. Our standard clinical practice is to start combined continuous OCPs on cycle day 1–3 of first menses following negative beta hCG and continue until completion of the OHS. All OHS are performed by board certified reproductive endocrinology and infertility specialists in a high-volume center. Furthermore, our institution had not yet implemented the use of mifepristone in medical management of EPL during the study period and thus used a misoprostol-only protocol, which may have contributed to slightly higher incidence of rPOC or need for surgical management following failed medical management. The current standard of care includes mifepristone in the medical treatment of EPL, given its improved efficacy in tissue expulsion, compared to an misoprostol alone protocol, when available (2, 24–26). This study only included patients who experienced an EPL after embryo transfer, therefore it remains unknown how the initiation of OCPs immediately following EPL in non-ART pregnancies would affect the incidence of rPOC. The data collected for this study relied on utilization of correct CPT codes to initially screen for patient eligibility. Errors in coding may have omitted patients during our study period. Lastly, we found there was a statistically significant difference in time between embryo transfer and subsequent OHS procedure for the two groups with longer time lapses in the non rPOC group, which may allow for resolution and passage of rPOC in some patients.

In conclusion, this cohort study did not find an association between the initiation of OCPs following EPL and rPOC, however, the retrospective design and modest sample size may have limited our ability to detect a clinically meaningful association. These findings provide preliminary evidence that OCP initiation does not increase the risk of rPOC, although larger prospective studies are still needed. Among available clinical factors, only history of prior uterine surgery before the embryo transfer cycle was associated with an increased risk of rPOC. This study found that common demographic and treatment factors, as well as initiation of OCPs after EPL following embryo transfer might not significantly increase the risk of rPOC. Future studies could also benefit from the standardization of protocols regarding the timeline between OCP initiation and office hysteroscopy to better understand the association with rPOC.

## Data Availability

The raw data supporting the conclusions of this article will be made available by the authors, without undue reservation.
