# Supplementary material for: Immediate initiation of oral contraceptive pills after an early pregnancy loss does not increase the risk of retained products of conception
Source: Front Reprod Health. 2026 Sep 11;8:1918673. doi: 10.3389/frph.2026.1918673 (PMC13612538; doi:10.3389/frph.2026.1918673)
Supplement: Supplementary file 1 [file Table1.docx]

Supplemental Table 1. Comparison between patients with OCP use and without OCP use

| **Characteristic** | **Use of OCP** | | **p-value^2^** |
| --- | --- | --- | --- |
|  | Yes  N = 55^1^ | No  N = 133^1^ |  |
| Age at transfer | 34.8 (32.2, 37.1) | 36.0 (32.4, 39.1) | 0.049 |
| Race |  |  | 0.5 |
| Caucasian | 40 (74.1%) | 108 (81.8%) |  |
| Asian | 9 (16.7%) | 13 (9.8%) |  |
| Other | 3 (5.6%) | 8 (6.1%) |  |
| Black or African-American | 2 (3.7%) | 3 (2.3%) |  |
| Missing | 1 | 1 |  |
| Ethnicity |  |  | 0.7 |
| Non-Hispanic | 51 (94.4%) | 125 (95.4%) |  |
| Hispanic | 3 (5.6%) | 6 (4.6%) |  |
| Missing | 1 | 2 |  |
| BMI | 25.0 (21.3, 29.0) | 26.0 (23.0, 30.0) | 0.12 |
| Cause of infertility |  |  | 0.8 |
| Ovulatory dysfunction | 19 (34.5%) | 57 (42.9%) |  |
| Male Factor | 17 (30.9%) | 31 (23.3%) |  |
| Other | 9 (16.4%) | 20 (15.0%) |  |
| Uterine factor | 7 (12.7%) | 18 (13.5%) |  |
| Unexplained | 3 (5.5%) | 7 (5.3%) |  |
| GYN diagnoses |  |  | 0.6 |
| None | 40 (72.7%) | 99 (74.4%) |  |
| Endometriosis | 8 (14.5%) | 17 (12.8%) |  |
| Endometrial Polyp | 4 (7.3%) | 9 (6.8%) |  |
| Fibroid | 2 (3.6%) | 8 (6.0%) |  |
| Other | 1 (1.8%) | 0 (0.0%) |  |
| History of Asherman’s syndrome | 4 (7.3%) | 4 (3.0%) | 0.2 |
| History of placenta accreta spectrum | 2 (3.6%) | 1 (0.8%) | 0.2 |
| History of uterine surgery | 34 (61.8%) | 89 (66.9%) | 0.5 |
| Number of prior uterine surgeries (D&C, MVA, CS) |  |  | 0.8 |
| 0 | 21 (38.2%) | 44 (33.1%) |  |
| 1 | 17 (30.9%) | 39 (29.3%) |  |
| 2 | 6 (10.9%) | 20 (15.0%) |  |
| >=3 | 11 (20.0%) | 30 (22.6%) |  |
| Uterine anomaly |  |  | >0.9 |
| None | 55 (100.0%) | 130 (97.7%) |  |
| Uterine septum | 0 (0.0%) | 1 (0.8%) |  |
| Bicornuate | 0 (0.0%) | 1 (0.8%) |  |
| Didelphys | 0 (0.0%) | 1 (0.8%) |  |
| Miscarriage Treatment |  |  | 0.14 |
| Surgical | 23 (41.8%) | 75 (56.4%) |  |
| Expectant | 23 (41.8%) | 37 (27.8%) |  |
| Medical | 9 (16.4%) | 21 (15.8%) |  |
| Time to OHS (days) | 106 (76, 159) | 106 (80, 148) | 0.8 |
| Retained products of conception | 13 (23.6%) | 29 (21.8%) | 0.8 |
| ^1^ Median (Q1, Q3); n (%)  ^2^ Wilcoxon rank sum test; Fisher’s exact test; Pearson’s Chi-squared test | | | |
